# Supplementary material for: Coherent spin qubit transport in silicon
Source: Nat Commun. 2021 Jul 5;12:4114. doi: 10.1038/s41467-021-24371-7 (PMC8257656; doi:10.1038/s41467-021-24371-7)
Supplement: Supplementary file 1 — Supplementary Information [file 41467_2021_24371_MOESM1_ESM.pdf]

Supplementary information:
Coherent spin qubit transport in silicon

J. Yoneda[1*†], W. Huang[1], M. Feng[1], C. H. Yang[1], K. W. Chan[1], T. Tanttu[1], W. Gilbert[1], R. C. C. Leon[1], F. E. Hudson[1], K. M. Itoh[2], A. Morello[1], S. D. Bartlett[3], A. Laucht[1], A. Saraiva[1], A. S. Dzurak[1*]

[1]*School of Electrical Engineering and Telecommunications, The University of New South Wales, Sydney, NSW 2052, Australia*

[2]*School of Fundamental Science and Technology, Keio University, Yokohama, Japan*

[3]*Centre for Engineered Quantum Systems, School of Physics, University of Sydney, Sydney, NSW 2006, Australia*

[†]Present address: *Tokyo Tech Academy for Super Smart Society, Tokyo Institute of Technology, Tokyo, 152-8552 Japan*

*Correspondence to: yoneda.j.aa@m.titech.ac.jp, a.dzurak@unsw.edu.au

Supplementary Note 1: Transport verification

Here we provide experimental evidence that the qubit is indeed transported between the two sites every time the detuning is ramped across the zero detuning point in the fidelity characterization experiments. We note that while it is possible to simulate the expected diabatic tunneling probability within the model using the extracted Hamiltonian parameters and the detuning ramp rate, the existence of noise and excited levels may introduce a subtlety to the dynamics in the experiment, in which case the wavefunction (or position) of the electron may not adiabatically follow the ground state of the electric potential controlled via gate voltages (on gates A and B with ε typically swept between +/-10 mV). Furthermore, the actual gate-voltage pulse shapes at the device end may well deviate from the voltage pulses generated in the room temperature circuit, as they are sent through filtered coaxial cables inside the dilution refrigerator (designed for a bandwidth of 80 MHz in our setup). We find it impracticable to estimate the distorted pulse shape in the presence of the transmission non-idealities, such as the standing wave modes, non-linear phase responses and nanosecond-scale inter-gate skews, inside the cryostat. Therefore, we design the following control experiment to verify that the qubit alternates between sites A and B for the same number of times as the number of applied transfer ramps, especially when we use the same pulse shape as in the fidelity characterization experiments.

The central idea of our control experiment is that the spin phase acquired under detuning pulses is very sensitive to the time the qubit spends in each site, due to the site-dependence of the qubit frequency (given by the slightly different g-factors, see the main text). We can precisely determine the spin precession rate for a given charge configuration and detuning from the tunneling spectroscopy result (see Fig. 2c and Supplenentary Fig. 2c). When ε is swept

between +/-10 mV, the difference in the phase precession rate is 32.4 MHz if the qubit switches its site following the (orbital) ground state as expected, with the dominant contribution coming from Δf_{AB}. In the meantime, if the qubit somehow remains in the same site, the precession rate would change by at most 1.2 MHz, since the Stark shift and the tunneling hybridization effect for these values of ε are much smaller. This means that during a 56 ns interval between the transfer ramps, the amount of spin phase accumulation will be around 3.6π in the case of adiabatic transfer as opposed to 0.13π in the absence of electron transfer.

In the actual experiment, we employ pulses with different odd numbers of ramps, n = 1, 3 and 5 (see Supplenentary Fig. 1a) and compare the qubit phases after the pulses. We henceforth denote the difference in the post-transfer spin phase between the pulses with the odd numbers of ramps, $n = i$ and i+2, by $\delta\phi_{i+2,i}$. The phase difference $\delta\phi_{i+2,i}$ will be approximately 3.6π when the electron does return to site A and spends roughly 56 ns longer time there (or, equivalently, 56 ns shorter time in site B) after the i-th ramp is completed. Conversely, if the electron fails to change its site, $\delta\phi_{i+2,i}$ will be much smaller (~0.13π).

We measure the fringes after the detuning ramps and quantify $\delta\phi_{i+2,i}$ from the difference in the fringe phase as a function of ε_1, where ε_1 is the specified value of ε at the ramp starting point (see Supplenentary Fig. 1a). By changing ε_1 in sufficiently small steps, this protocol allows us to evaluate $\delta\phi_{i+2,i}$ larger than 2π experimentally (with no ambiguity given $\delta\phi_{i+2,i} \approx 0$ for ε_1= +10 mV). It is important that the microwave phases of the two ESR pulses in this Ramsey-type sequence (applied at different sites and thus with different tones) be defined consistently for different numbers of ramps. The fringes obtained for various values of ε_1 are exemplified in Supplenentary Fig. 1b-f. Figure S1g plots the extracted values of $\delta\phi_{3,1}$ and $\delta\phi_{5,3}$ as a function of ε_1. The two traces agree well, suggesting the reliability of the phase measurement protocol and the consistency in the trajectories as n is incremented. When ε_1 is large (e.g. point f), the phase difference is constantly small as the electron is not shuttled between sites and stays in site B. At around ε_1= 2 mV (point e), the spin phase starts to pick up the tunneling hybridization effect around the anticrossing. Around ε_1 = -10 mV (point b), $\delta\phi_{i+2,i}$ reaches 3.6π, which can only be accounted for by a decrease in time spent in site B by ~56 ns upon increasing n by 2, indicating that following the i-th ramp, the qubit moves from site B to A (the (i+1)-th ramp) and, after dwelling for ~56 ns in site A, goes back to site B (the (i+2)-th ramp). We therefore conclude that for ε_1 = -10 mV, the same value that was used for the transfer fidelity measurements, the electron consistently moves between the sites for n times.

The data is even more compelling when we compare them with the calculation based on the qubit spectrum. The traces are already well explained by assuming that the actual qubit detuning is identical to the specified detuning pulse and that the dynamics is completely adiabatic (see the grey solid curve in Supplenentary Fig. 1g). For illustration purposes, we plot the results for two other transfer functions between the specified detuning pulse and the actual

qubit detuning, which yield a slightly better alignment with the data, compared to the unfiltered case: a Butterworth filter (6th-order 40 MHz lowpass) and a Chebyshev filter (type 1, 1st-order bandstop with a 25-50 MHz stopband and a 1dB ripple level). The trajectories of the qubit detuning and frequency for ε_1 = -10 mV are also shown for individual cases in Supplenentary Fig. 1h-m. In all cases, the simulations verify that the qubit is transported between sites, further reinforcing our conclusion.

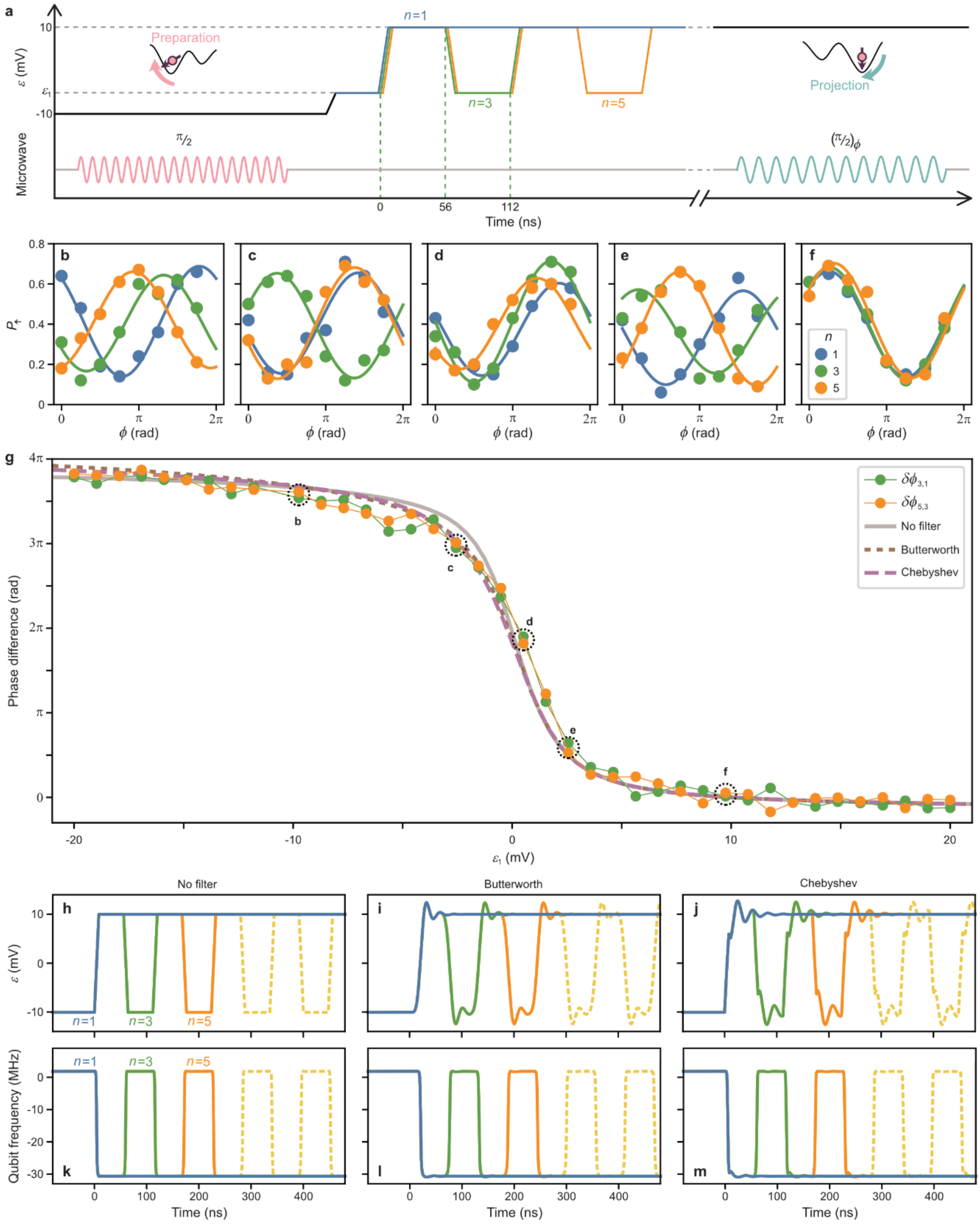

Supplementary Fig. 1. Transport verification. (a) Pulse sequences used for transport verification. Laterally offset for clarity. **(b)-(f)** Ramsey fringes observed for various ε_1 (specified in (g)) along with fit curves from which to extract the phase. **(g)** Measured and calculated phase differences $\delta\phi_{i+2,i}$ as a function of ε_1. For illustration, we employ three transfer functions between the specified detuning pulse and the actual qubit detuning. Data points with small (<0.04) fringe amplitudes are omitted. **(h)-(j)** Qubit detuning trajectories for exemplified transfer functions. **(k)-(m)** Corresponding trajectories of the qubit frequency.

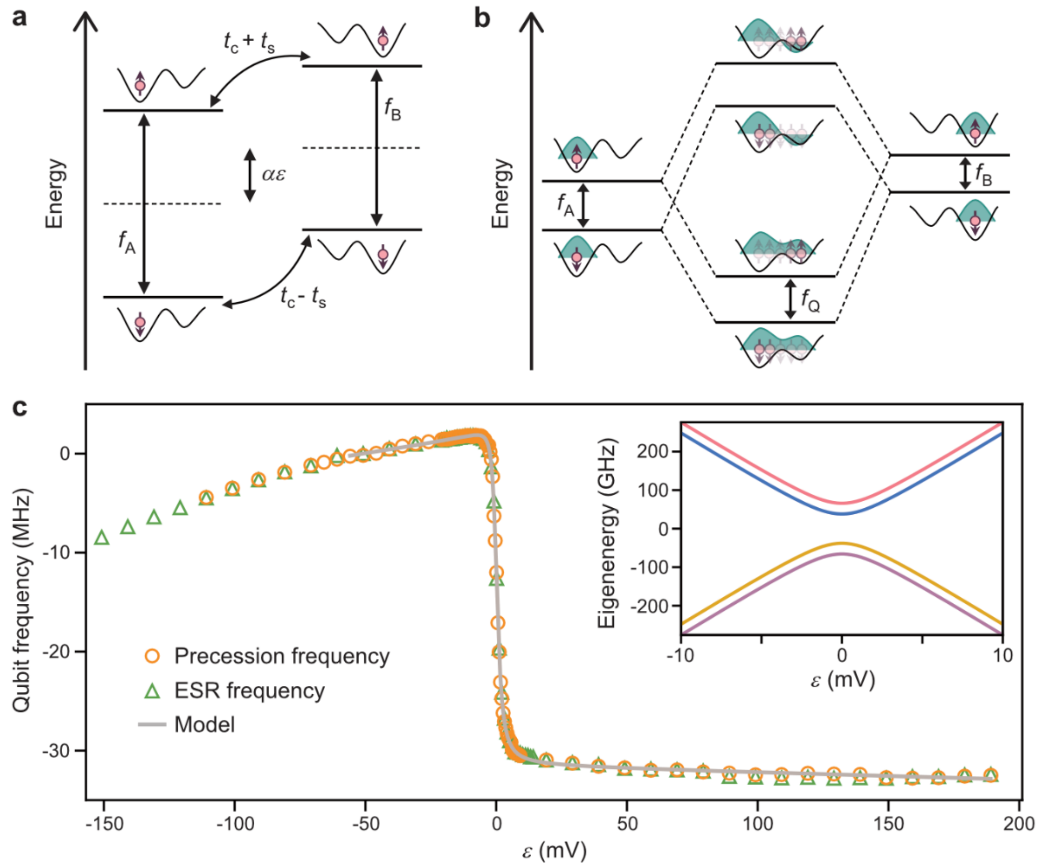

Supplementary Fig. 2. Qubit spectrum modelling. (a) Illustration of the Hamiltonian terms used in the model. **(b)** Energy diagram showing the tunnelling hybridization. The left and rightmost columns depict the bare energy levels in individual sites in the absence of tunnelling. Energy levels in the middle column result from tunnelling hybridization. f_Q gives the qubit frequency. **(c)** Wide-span qubit spectrum and the fit to the model. The finite slopes in the far detuned region are due to the Stark shift. The inset shows the eigen-energies calculated from the model.

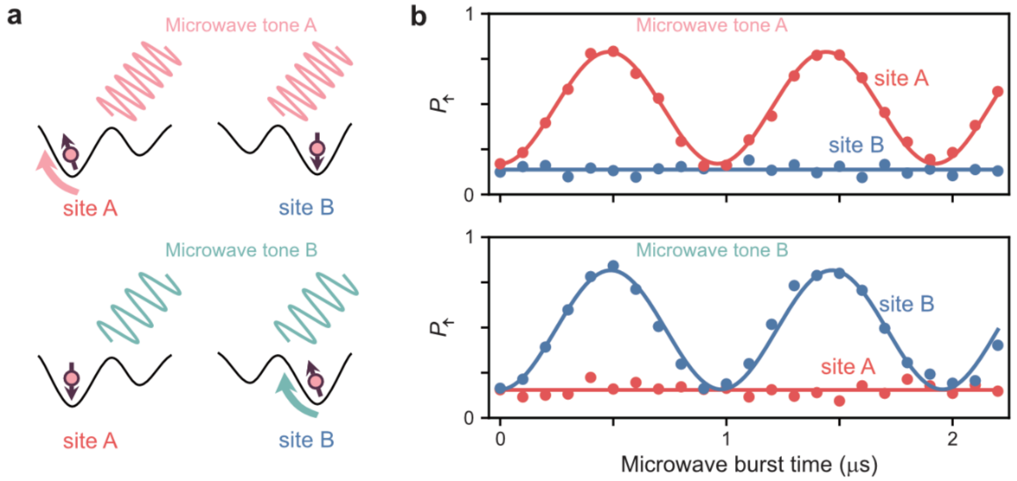

Supplementary Fig. 3. Site-dependent qubit response to a fixed ESR control tone. (a) Schematics showing the site-dependent qubit response to ESR microwave pulses. The qubit rotates only when its site matches the microwave tone used. **(b)** Rabi oscillations observed by turning on and off the ESR drive by switching the qubit site.

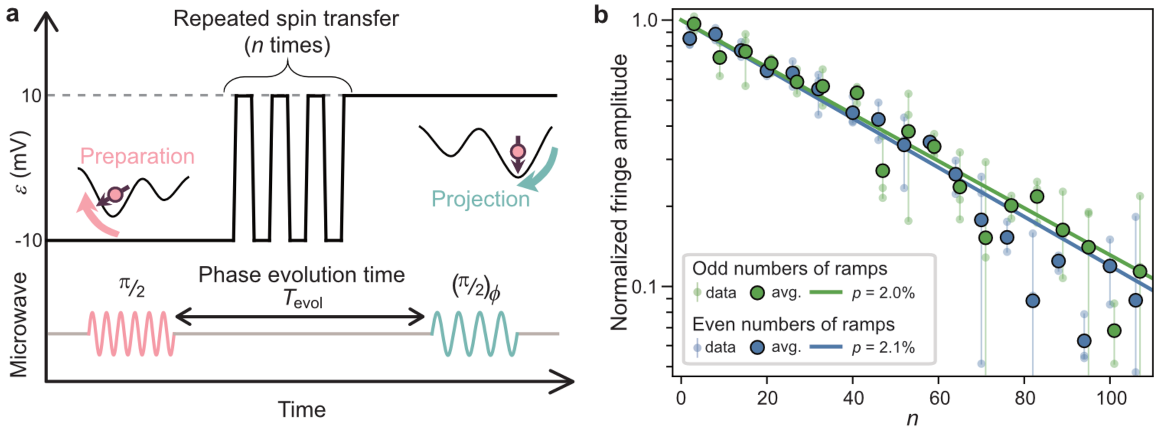

Supplementary Fig. 4. Coherent transfer fidelity measurement with odd numbers of ramps. (a) Pulse schematics for odd numbers of ramps. The projection ESR pulse is applied while the qubit is in site B. **(b)** Comparison of the fringe amplitude decays for the cases of odd (green) and even n (blue, same data as in Fig. 4c). The phase evolution time is varied in both cases.

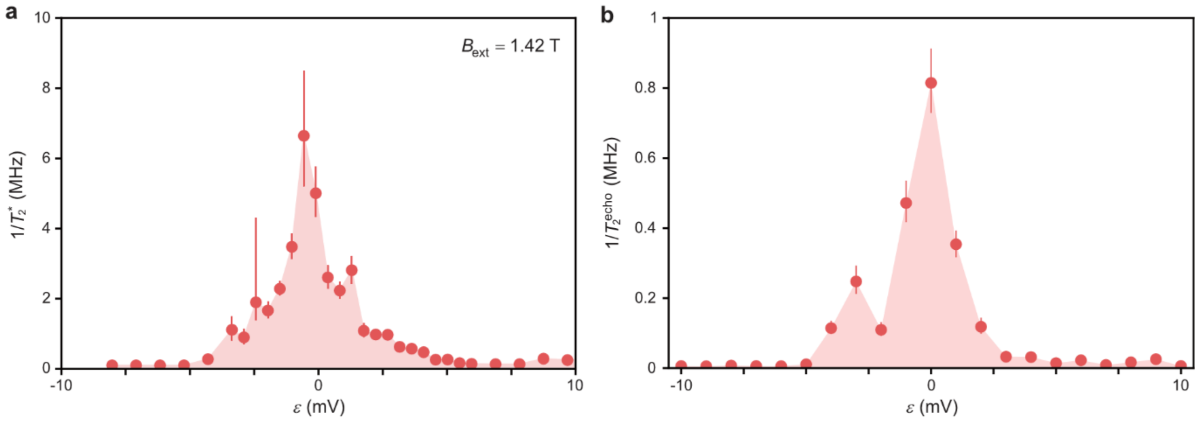

Supplementary Fig. 5. Coherence times measured near zero detuning. (a) The inverse of T_2^* plotted as a function of ε. In this data set, the external field applied (1.42 T) is larger by a factor of 1.42, and Δf_{AB} is enhanced to 49.4 MHz. The qubit is expected to be proportionally more susceptible to charge noise around $\varepsilon = 0$. The device gate-voltage configuration is slightly different as well. **(b)** The inverse of Hahn echo time T_2^{echo} as a function of ε. The data shows that dephasing around $\varepsilon = 0$ is mostly refocused by an echo sequence (note the 10-fold difference in the *y*-scale). Error bars represent the 1σ confidence intervals of the coherence times.

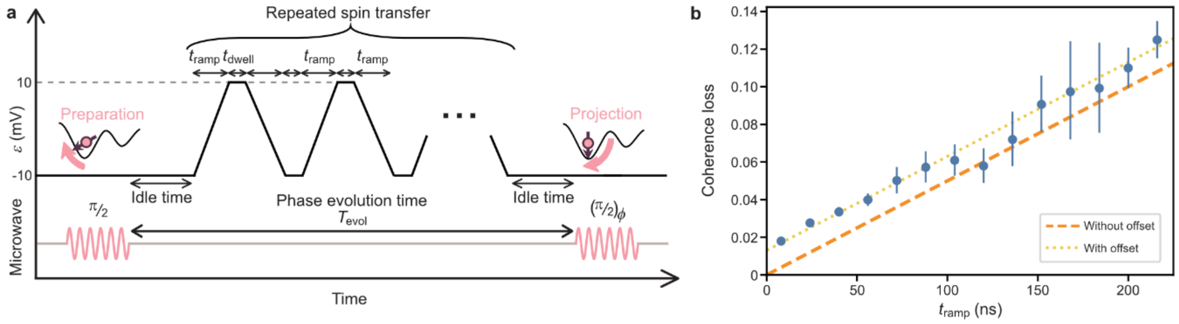

Supplementary Fig. 6. Ramp time dependence. (a) Pulse schematic used for ramp time dependence measurement. We change the ramp time denoted by t_{ramp}, while fixing the dwell time to 48 ns. The idle time before and after the repeated transfer ramps is adjusted to keep T_{evol} constant. **(b)** Coherence loss as a function of t_{ramp}. The orange dashed (yellow dotted) line shows a linear guide to the eye without (with) a 1.3 % offset. Error bars represent the 1σ confidence intervals of coherence loss.
